# Characterization of Neoantigen Load Subgroups in Gynecologic and Breast Cancers

**DOI:** 10.3389/fbioe.2020.00702

**Published:** 2020-07-13

**Authors:** Yue Zhu, Xiaowei Meng, Xinjia Ruan, Xiaofan Lu, Fangrong Yan, Fei Wang

**Affiliations:** State Key Laboratory of Natural Medicines, Research Center of Biostatistics and Computational Pharmacy, China Pharmaceutical University, Nanjing, China

**Keywords:** gynecologic and breast cancer, neoantigen load, immune infiltrate, intratumor heterogeneity, immunotherapy

## Abstract

**Objective:**

Although gynecologic and breast (Pan-Gyn) cancers share a variety of similar characteristics, their response to immunotherapy is different. Immune checkpoint inhibitor therapy is not effective in all patients, while neoantigen load (NAL) may be a predictive biomarker. However, the selection of a NAL cutoff point and its predictive effect remain to be elucidated.

**Methods:**

We divided 812 Pan-Gyn cancer samples from The Cancer Genome Atlas into three groups based on 60 and 80% of their load percentile. We then correlated the identified NAL subgroups with gene expression, somatic mutation, DNA methylation, and clinicopathological information. We also characterized each subgroup by distinct immune cell enrichment, PD-1 signaling, and cytolytic activity. Finally, we predicted the response of each subgroup to chemotherapy and immunotherapy.

**Results:**

Across Pan-Gyn cancers, we identified three distinct NAL subgroups. These subgroups showed differences in biological function, genetic information, clinical variables, and immune infiltration. Eighty percent was identified as a meaningful cutoff point for NAL. In all patients, a higher NAL (top 20%) was associated with better overall survival as well as high immune infiltration and low intra-tumor heterogeneity. Furthermore, an interesting lncRNA named AC092580.4 was found, which was associated with two significantly different immune genes (CXCL9 and CXCL13).

**Conclusions:**

Our novel findings provide further insights into the NAL of Pan-Gyn cancers and may open up novel opportunities for their exploitation toward personalized treatment with immunotherapy.

## Introduction

In recent years, cancer immunotherapy, especially immune checkpoint inhibitor (ICI) treatment, has revolutionized the traditional treatments of patients with advanced tumor. Antibodies targeting CTLA-4 or PD-1/PD-L1 are effective in treating a variety of malignant tumors (Callahan et al., 2016). However, durable benefits are limited to a minority of patients. The biological mechanisms that drive the individual heterogeneity of these responses are not fully understood, but are important for the design of personalized immunotherapy strategies. Recently, some phase 3 clinical trials reported negative results for both non-selective patients and selected groups, emphasizing the importance of better predictive biomarkers for clinical demand (Carbone et al., 2017; Powles et al., 2018). Early findings have indicated that PD-L1 immunohistochemistry, peripheral blood markers, several relative gene expression signatures, and T cell receptor clonality may be associated with clinical response (Gibney et al., 2016). For example, tumor-associated macrophages (TAM) and regulatory T cells (Tregs) are associated with tumor-promoting function (Nishikawa and Sakaguchi, 2014), while CD8^+^ T cells are associated with improved clinical outcomes and immunotherapeutic responses (Tumeh et al., 2014). The antitumor activity of antigen-specific CD8^+^ T cells may be the basis for the efficacy of immune checkpoint blocking therapy since the amount and activity of CD8^+^ T cells increase with these drugs (Rizvi et al., 2015). Conversely, a low T cell density is associated with poor prognosis (Galon et al., 2007). Additionally, a correlation between a high mutation load and clinical benefit to immune checkpoint blockade was observed in a small cohort of patients with melanoma and lung and colon cancers (Snyder et al., 2014; Le et al., 2015; Rizvi et al., 2015; Hugo et al., 2016). Moreover, a high tumor mutational burden (TMB) usually significantly correlates with higher tumor-infiltrating lymphocyte (TIL) levels (Thomas et al., 2018), and TMB has been proven to be a predictive biomarker for clinical benefit after immunotherapy (Samstein et al., 2019).

Nowadays, with the development of new sequencing technologies, specialized computational methods, and human leukocyte antigen (HLA) binding predictions, neoantigen has been utilized. These neoepitopes with specific amino acid sequence variations generated by cancer somatic mutations can be recognized by the immune system. Generally, patients with high TMB have more neoantigens. However, it is unclear whether a high neoantigen load (NAL) is robustly predictive of clinical benefit across diverse human tumors.

Gynecologic and breast (Pan-Gyn) cancers share several kinds of similar characteristics: the Müllerian duct’s development, the influence of female hormones, and the special gynecologic oncology effect (Mullen and Behringer, 2014). Recently, similarities in molecular characteristics have been found in Pan-Gyn cancers in a comprehensive pan-cancer analysis study (Berger et al., 2018; Hoadley et al., 2018). These cancers have been proven to be highly immune-infiltrating tumors in various clinical and genomic studies (Bregar et al., 2017). However, the mechanisms of malignant tumor immunity infiltration and immune response to treatment are still poorly understood.

In this study, we investigated the association between NAL and overall survival across the following five The Cancer Genome Atlas (TCGA) cancer types: breast carcinomas (BRCA), uterine cervical carcinomas (CESC), ovary carcinomas (OV), endometrial carcinomas (UCEC), and uterine carcinosarcomas (UCS). They represent the most frequent and aggressive gynecologic cancers. To better understand the complex impact of neoantigens, we further comprehensively characterized the NAL subgroups in a multiple omics view including somatic mutation, gene expression, DNA methylation, and long non-coding RNA expressions in these five gynecologic cancers. We characterized the subgroups by immunity infiltration state, intra-tumor heterogeneity (ITH), PD-L1 immune blocking point inhibitor, and other immune signatures. We also predicted the response of each subgroup to immunotherapy and chemotherapy. The present research can provide necessary biological information for NAL, guidance on personalized immunotherapy options, and decision on patients’ management.

## Materials and Methods

### Patients Tumor Samples

The mutation annotation file (MAF) files containing Pan-Gyn cancers’ somatic mutation information and DNA methylation beta value were obtained from The Cancer Genome Atlas Project (TCGA) pan-cancer analyses data portal^1^. Transcriptomic sequencing (RNA-Seq) raw count data of the Pan-Gyn cancers with 2,199 tumor samples, including 1,049 BRCA, 186 CESC, 419 OV, 488 UCEC, and 57 UCS, were downloaded from the GDC data portal^2^. Nine hundred and thirty one immune-related Pan-Gyn samples were selected with *p* < 0.05 by the CIBERSORT algorithm (Newman et al., 2015). The corresponding clinical and pathologic information files were obtained from Firehose^3^. The 4,165 gynecologic tumor-specific potential neoantigens predicted by NetMHCpan 2.8 were available from TSNAdb^4^ (Hoof et al., 2009; Wu et al., 2018).

### Neoantigen Load Assessment

The MAF file with 812 Pan-Gyn cancer samples was filtered by tumor-specific neoantigens. The total number of neoantigens identified was normalized to the exonic coverage sequenced. The R package “maftools” was used to compute the Pan-Gyn NAL with the MAF file (Mayakonda et al., 2018). Neoantigen load cutoffs of 60 and 80% were selected based on the different immune states, obtaining 163 samples as the neoantigen load-high (NAL-H) group, 161 samples as the neoantigen load-middle (NAL-M) group, and 488 samples as the neoantigen load-low (NAL-L) group.

### RNA Analysis

The Ensembl ID for genes was annotated in GENCODE27 to obtain gene symbol names. The protein coding genes [messenger RNA (mRNA)] and long non-coding RNA (lncRNA) were selected. Raw count data were then converted into FPKM (the fragments per kilobase of exon per million fragments mapped) values for analysis. To reduce noise, we filtered out low-expression genes with FPKM values below 1 in at least 90% of the samples. Batch effect removal was performed by the R package “combat.” Differential expression analysis among the NAL subgroups was performed by the R package “limma” with the standard comparison mode. The significantly differentially expressed genes were obtained with a false discovery rate (FDR) < 0.05 and fold change greater than 2 for overexpression or less than 0.5 for down-expression. Gene Ontology (GO) annotation was then performed using the R package “clusterProfiler” to characterize the subgroups according to the differentially expressed mRNAs. The correlation between the lncRNAs and mRNAs was computed, and differentially expressed lncRNAs were filtered with a correlation higher than 0.6. lncRNA functions were predicted with their highly correlated genes using gene set enrichment analysis (GSEA) in the R package “clusterProfiler” (Yu et al., 2012).

### Integrative Analysis of DNA Methylation and mRNA Expression

We performed integrative analysis among the DNA methylation and mRNA expression to explore epigenetically silenced or activated genes (epi-silenced/activated genes). Specifically, we chose methylation probes that were differentially expressed in the NAL subgroups and excluded the correlations between probes and mRNAs. Differentially methylated analysis was performed by the R package “limma” on defined subgroups. The significantly different methylation probes were obtained with FDR < 0.05. We combined the differential expression information with the differential methylation results by correlation analysis. The methylation probes of interest were filtered with correlation higher than 0.6.

### Mutation Analysis

MutSigCV was used to infer significant tumor-mutated genes (*q* < 0.05) with default parameters (Lawrence et al., 2013). Significantly different mutations among neoantigens load subgroups were obtained using the R package “limma” (FDR < 0.05). According to the hg19 human reference genome^5^, we also analyzed 30 mutation signatures with the MAF file and compared the mutation signatures among the identified subgroups.

### Chemotherapeutic Response Prediction

We predicted the chemotherapy response for each sample based on the largest public pharmacogenomics database, the Genomics of Drug Sensitivity in Cancer (GDSC). Three commonly used and three other useful chemicals were selected, namely, cisplatin, docetaxel, paclitaxel, etoposide, vinorelbine, and gemcitabine. The prediction process was performed by the R package “pRRophetic,” in which the IC_50_ of the sample was estimated by ridge regression and the prediction accuracy was evaluated by 10-fold cross-validation based on the GDSC training set. All parameters were set by the fault value, with “combat” for batch effect removal and the expression of repeated genes summarized as the average (Geeleher et al., 2014).

### Statistical Analysis

All statistical tests were performed by R/3.6.1, using χ^2^ or Fisher’s exact test for the categorical data, two-sample Wilcoxon test (Mann–Whitney test) for continuous data, and Kaplan–Meier curve of log-rank test and Cox regression for the hazard ratio (HR). Survival analysis was executed by using the R package “survival.” Log-rank test was used to estimate the *p* value. Fisher’s exact independence test was used to statistically test the association between the categorical clinical information and the identified neoantigen subgroups. For all statistical analyses, a *p* value less than 0.05 was considered statistically significant. All statistical modeling and visualization were performed using the R language.

## Results

### Overview of Sample Selection and Subgroup Identification

Among all 2,199 Pan-Gyn tumor samples, 931 significant immune-related samples were diagnosed (*p* < 0.05) by the CIBERSORT algorithm. Based on the mutation information of the neoantigens in the MAF file, 812 samples were selected, including 441 BRCA, 71 CESC, 96 OV, 180 UCEC, and 24 UCS (Supplementary Figure S2). The 812 Pan-Gyn samples with full survival and clinic pathological information were retained for downstream analysis.

Four thousand one hundred and sixty-five potential neoantigens from the TSNAdb gene sites were selected by integrating the information obtained above. According to the MAF file, the mutation information on the 4,118 neoantigen gene sites were kept finally. We used the R package “maftools” to describe the landscape of the filtered MAF files and compute the NAL (Supplementary Figures S1, S2). Selecting 60 and 80% as the NAL cutoffs, 163 samples as the NAL-H group, 161 samples as the NAL-M group, and 488 samples as the NAL-L group were identified.

### Different Functional Pathways Among the Neoantigen Load Subgroups

**FIGURE 1:**
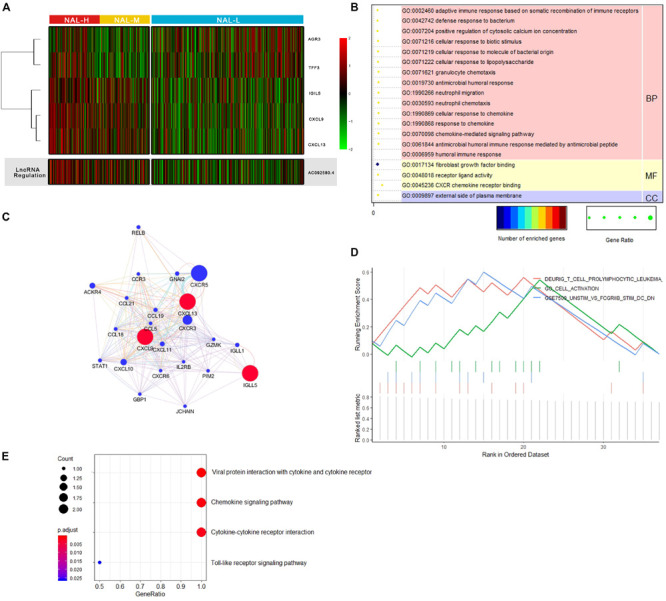
**(A)** Heat map of the differentially expressed genes and an lncRNA of interest among the subgroups. **(B)** Gene Ontology (GO) functional annotation for the differentially expressed genes from the cellular component (*CC*), molecular function (*MF*), and biological process (*BP*) aspects. **(C)** Interaction network (generated by GeneMANIA) by three differentially expressed genes. **(D)** GESA results for AC092580.4 related genes. **(E)** Kyoto Encyclopedia of Genes and Genomes (KEGG) results for three interesting differentially expressed genes.

We firstly used “combat” to remove the batch effect in the expression profiles (Supplementary Figure S2). Then differential expression analysis identified five significantly dysregulated genes (CXCL9, CXCL13, IGLL5, AGR3, and TFF3) with a threshold of FDR < 0.05 and absolute log2(fold change) > 1 (Supplementary Table S1). Among them, three genes (CXCL9, CXCL13, and IGLL5) were all upregulated for the NAL-H and NAL-M subg roups compared with the NAL-L subgroup (Figure 1) and were involved in immunological processes such as T cell trafficking, B lymphocyte migration, and antigen binding. Gene Ontology annotation for these genes indicated enrichment of the immune-related terms (Figure 1 and Supplementary Table S2), such as humoral immune response (FDR = 0.001), chemokine-mediated signaling pathway (FDR = 0.002), and adaptive immune response based on somatic recombination of immune receptors built from immunoglobulin superfamily domains (FDR = 0.008). The Kyoto Encyclopedia of Genes and Genomes (KEGG) pathway analysis denoted that these genes were involved in cytokine–cytokine receptor interaction, Toll-like receptor signaling pathway, and leukocyte transendothelial migration (Figure 1). The genetic interaction network of the three differentially expressed genes was generated by GeneMANIA. Different line and node colors in Figure 1 represent different types of interactions and different immune-related functions.

We compared the correlation between the lncRNA genes and the three genes above. Interestingly, we found a lncRNA named AC092580.4, which had strong correlations with CXCL9 and CXCL13 and was significantly upregulated in the NAL-H and NAL-M subgroups (Figure 1). To better understand its function, we selected 37 genes that were highly related with AC092580.4 as a gene set (Supplementary Table S3) and used GSEA to analyze this pre-ranked gene list (Figure 1). Nineteen terms were enriched under this lncRNA (Supplementary Table S4), such as immune-related cells [e.g., CD8 T cells, CD4 T, and cells and natural killer (NK) cells] and pathways (e.g., lymphocyte activation, leukocyte activation, cell death, and positive regulation of immune system process).

### Somatic Mutation Landscape of the Neoantigen Load Subgroups

**FIGURE 2:**
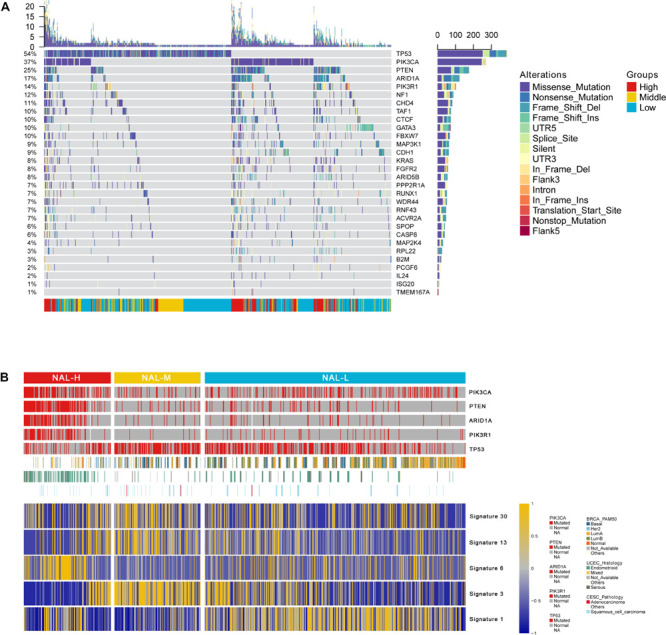
**(A)** Oncoprint shows the somatic mutation landscape of MutSigCV detecting differentially mutated genes among the subgroups. **(B)** Heat map of the differential mutation signatures among the subgroups.

Under a stringent threshold of *q* < 0.05, MutSigCV identified 30 significantly mutated genes (SMGs) among all Pan-Gyn tumor samples, including 18 neoantigen gene sites of greater interest (Figure 2 and Supplementary Table S5). All these mutated genes differed in the frequency of somatic mutations among the NAL subgroups (FDR < 0.05). For the top 5 most frequently mutated genes [TP53 (48% of the samples mutated), PIK3CA (33%), PTEN (22%), ARID1A (15%), and PIK3R1 (12%)], we described their distributions in the NAL subgroups (Figure 2). These five genes have been reported in previous papers (Berger et al., 2018). However, among all the TCGA gynecologic landmark papers, there were no previous reports of ACVR2A (Supplementary Table S5), one of the 18 significantly different neoantigen mutations (Berger et al., 2018). ACVR2A is a member of the transforming growth factor beta superfamily that plays a role in pathways associated with tumor progression and suppression (Ikushima and Miyazono, 2010).

Here, we also evaluated 30 mutation signatures to better understand the complex mutational processes. Mutational signatures provide insight into the mechanisms of tumor development and contribute to patient treatment decisions (Helleday et al., 2014). Among them, five significantly different signatures were obtained, including signature 1, signature 3, signature 6, signature 13, and signature 30 (FDR < 0.05) (Figure 2). NAL-H was enriched in signature 6, which is related to a DNA mismatch repair defect that suggests sensitivity to checkpoint inhibitors (Berger et al., 2018). NAL-M was enriched in signatures 3, 13, and 30. Signature 3 is closely associated with germline and somatic BRCA1 and BRCA2 mutations in breast, pancreatic, and ovarian cancers. Signature 13 indicates AID/APOBEC family activity of cytidine deaminase. Signature 30 has been observed in a small subset of breast cancers. NAL-L was enriched in signature 1, indicating that the endogenous process was initiated by the spontaneous demineralization of 5-methylcytosine.

### Association Between Neoantigen Load Subgroups and the Clinical Outcomes

**FIGURE 3:**
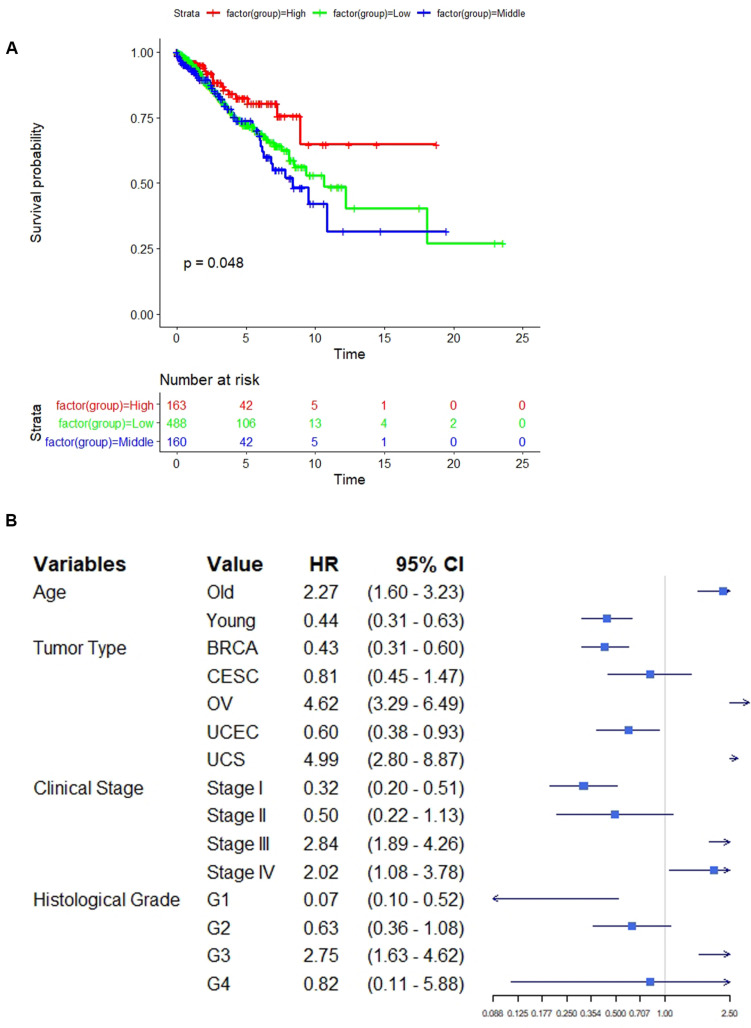
**(A)** Kaplan–Meier curves of the overall survival time for comparing the three subgroups. **(B)** The forest plot shows some clinical variables affecting patients’ overall survival.

As expected, NAL-H showed the best survival compared with NAL-M and NAL-L (*p* = 0.048, HR = 0.578, 95% CI = 0.361–0.924) (Figure 3). We then compared the differences in the clinical covariates among the identified subgroups (Table 1). Based on the clinical information of patients, we examined multiple variables including age, gender, tumor stage, tumor type, clinical stage, histological grade, and menopause status. For the age of patients belonging to the continuous variable, all samples were divided into two groups with a cutoff point equal to 55. For other categorical variables, we used their classification information for group comparison. Four significantly different variables were obtained, including age, tumor type, clinical stage, and histological grade. We then converted them into latent variables and performed univariate Cox regression to determine whether these parameters affected patient outcome (Figure 3). Older patients had an increased risk of poor prognosis (HR = 2.27). BRCA, CESC, and UCEC patients had better prognosis (HR = 0.43, 0.81, and 0.60, respectively). Early tumor stage and a lower histological grade could favor the prognosis of patients (stage I: HR = 0.32; stage II: HR = 0.50; G1: HR = 0.07; G2: HR = 0.63).

**TABLE 1:** Comparison of cohorts according to clinical parameters.

**Index**	**Group**	**Subgroups**			***p* value**
		**NAL-H**	**NAL-M**	**NAL-L**	
Age (years)	≤55	103	106	271	0.0276*
	>55	58	55	217	
Gender	Female	163	161	484	0.4797
	Male	0	0	4	
Tumor stage	Stage I	5	10	56	0.7686
	Stage II	28	52	191	
	Stage III	7	14	61	
	Stage IV	0	1	11	
Tumor type	BRCA	40	78	323	0.0005***
	CESC	17	20	34	
	OV	13	45	38	
	UCEC	91	16	73	
	UCS	2	2	20	
Clinical stage	Stage I	74	20	79	1.42e−105***
	Stage II	11	11	12	
	Stage III	32	47	57	
	Stage IV	5	4	15	
Histological grade	G1	18	3	18	0.0387*
	G2	28	15	37	
	G3	71	62	88	
	G4	4	1	1	
Menopause status	Premenopausal	24	24	87	0.5402
	Postmenopausal	105	79	295	
	Indeterminate	10	4	32	

*p < 0.05; **p < 0.01; ***p < 0.001.

### Immune Infiltration Differences Associated With the Neoantigen Load Subgroups

**FIGURE 4:**
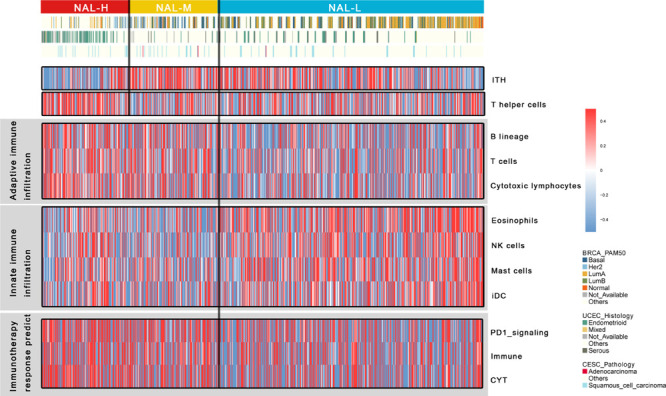
Heat map of gynecologic cancers’ immune infiltrate state. High and low enrichment scores are presented in *red* and *blue*, respectively.

We evaluated several immune-related signatures to gain further immunologic insights on these subgroups by single-sample GSEA. Interestingly, there were significant differences in immune enrichment among the three subgroups. As shown, NAL-H and NAL-M had significantly higher degrees of adaptive immune infiltration in T cells, B cells, and cytotoxic lymphocytes, while NAL-L enriched with innate immune infiltration in eosinophils, NK cells, mast cells, and interdigitating cells (iDC), and the differences within these subgroups need to be further studied (Figure 4). Further research is needed on the causes of the different immune infiltrations among subgroups.

Specifically, we noticed that NAL-H had significantly lower ITH, which corresponded with early findings that a low ITH was an important determinant of good response to checkpoint therapy (Wolf et al., 2019). Intra-tumor heterogeneity is independent of tumor mutation burden and can affect tumor invasiveness and immunity. A high-ITH tumor has a strong immunosuppressive tumor microenvironment. Moreover, NAL-H was significantly enriched in T helper cells. A recent paper reported that the activity of tumor antigen-specific CD8^+^ and CD4^+^ T cells could induce antitumor response in immunotherapy. The immune response of T helper cells is that CD4^+^ T cells can recognize MHC II antigen, which plays an important role in anticancer activity (Alspach et al., 2019).

### Differential Response With Chemotherapy and Immunotherapy in Subgroups

To evaluate the traditional chemotherapeutic response of the NAL subgroups, we trained a predictive model using the GDSC cell line dataset by ridge regression and assessed its satisfactory prediction accuracy by 10-fold cross-validation. We estimated the IC_50_ for each sample in the Pan-Gyn cancers based on a predictive model of six chemical drugs (Supplementary Figure S3). We identified significant differences in the estimated IC_50_ for these subgroups of all these chemotherapeutic drugs (cisplatin: *p* = 0.0005; paclitaxel: *p* = 0.0057; docetaxel: *p* = 0.0001; etoposide: *p* = 0.0076; vinorelbine: *p* = 0.0032; and gemcitabine: *p* = 0.0010). NAL-H and NAL-M could be more sensitive to all six drugs than NAL-L. Specifically, NAL-M had a significantly sensitive response to docetaxel, etoposide, and paclitaxel. NAL-M was composed of most basal-like breast carcinomas (BLBC). This finding was consistent with the report that BLBC has a relative sensitivity to chemotherapy, which may provide opportunities for optimizing treatment (Bertucci et al., 2012).

Considering that immunotherapy has revolutionized the treatment of patients with cancers, we used some immune signatures to evaluate the ICI treatment response. Early studies have shown that a high immune cytolytic activity (CYT) is significantly associated with significant pan-cancer survival benefits (Rooney et al., 2015) and effectively corresponds to anti-CTLA-4 and anti-PD-L1 immunotherapy (Ji et al., 2012; Herbst et al., 2014). We found significant high CYT in NAL-H and NAL-M, corresponding to the upregulation of PD-1 signaling signature genes (Figure 4). In conclusion, the presence of CYT, high immune infiltration, and PD-1 signaling may suggest that NAL-H and NAL-M have good response to immunotherapy, especially ICI treatment.

### Further Exploration of the Difference Between NAL-H and NAL-M

**FIGURE 5:**
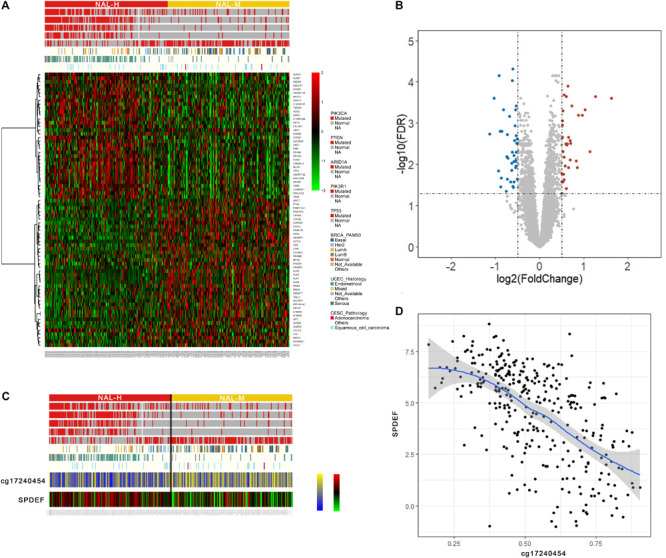
**(A)** Heat map of the differentially expressed genes between the neoantigen load—high (NAL-H) and neoantigen load—medium (NAL-M). **(B)** The volcano plot shows the differential genes between groups. **(C)** Heat map of a gene of interest and its highly related methylation probe. **(D)** Correlation of a gene of interest and its highly related methylation probe.

Based on the above analysis, NAL-H and NAL-M were similar in many aspects, including differentially expressed genes, levels of immune infiltration, response to treatment, etc. In order to further understand the differences between these two subgroups, differential expression analysis identified 74 significantly dysregulated genes with a threshold of FDR < 0.05 and absolute log2(fold change) > 0.5, including 34 overexpressed and 40 down-expressed genes (Figure 5). The GSEA for these genes indicated enrichment of 15 terms, and three of them caught our interest, including ESR1 targets down in NAL-H (FDR = 0.0078), ESR1 up in NAL-H (FDR = 0.0078), and martens tretinoin response up in NAL-M (FDR = 0.014) (Supplementary Table S6). Gene Ontology annotations of ESR1 included DNA-binding transcription factor activity and identical protein binding. A volcano plot was used to display the fold difference of these genes between the subgroups (Figure 5).

We also compared the correlation between the significantly different DNA methylation probes and the 74 genes above. Specifically, we found an interesting methylation probe named cg17240454, which had strong negative correlation with SPDEF (Figure 5).

## Discussion

Good predictive biomarkers are needed to predict survival after immunotherapy in Pan-Gyn cancers. In this study, to investigate the ability of NAL as a predictive biomarker, we performed an integrative, multi-omics data analysis of the TCGA Pan-Gyn cancers based on 812 samples. We studied the relationship between the NAL subgroups and overall survival, the characterization of the NAL subgroups with multiple omics data, the estimation of the immune infiltrate status from several immune signatures, and the ability to predict the outcome of immunotherapy and chemotherapy. As NAL provides important information for clinical immunotherapy selection, we attempted to explain the biological mechanism behind it.

We selected 80 and 60% as the NAL cutoff points, which divided the 812 samples into three groups: NAL-H, NAL-M, and NAL-L. Three significantly overexpressed genes (CXCL9, CXCL13, and IGLL5) in NAL-H and NAL-M were found, and the GO annotation and KEGG analysis indicated that these genes were enriched in immune-related terms and pathways. The TNF-α network activates macrophages and B cells, leading to strong upregulation of the gene expressions of several chemokines, especially CXCL13 (Shu et al., 2012). The IFN-γ network causes CXCL9 to be upregulated in different cells, including macrophages (Shu et al., 2012). Lymphocyte chemoattractants (CXCL9 and CXCL13) as immune-activating factors revealed a high immune state of NAL-H and NAL-M, and they were highly significant predictors of therapy response. Interestingly, we found an lncRNA named AC092580.4, which was significantly correlated with CXCL9 and CXCL13 genes and was significantly upregulated in NAL-H and NAL-M. This lncRNA may promote the expressions of CXCL9 and CXCL13. We further studied the function of AC092580.4 and found that it had an upward regulation effect on some immune-related cells and pathways.

We used several immune signatures to compare the differences of the immune infiltrate status among subgroups. Similarly, we noticed that NAL-H and NAL-M were significantly enriched in higher degree of adaptive immune infiltration, including T cells, B cells, and cytotoxic lymphocytes, while NAL-L was enriched in innate immune infiltration, including eosinophils, NK cells, mast cells, and iDC. To better understand the response of treatment, we evaluated subgroup responses to six traditional chemotherapy drugs. NAL-H and NAL-M could be more sensitive to all six drugs than NAL-L, and the higher enrichment of CYT, immune infiltration, and PD-1 signaling might suggest that NAL-H and NAL-M had good response to immunotherapy. Both NAL-H and NAL-M appeared to respond well to chemotherapy and immunotherapy. Based on the above analysis, it seems that the 60% cutoff point of NAL can effectively distinguish the different immune status among Pan-Gyn cancers.

However, NAL-H had a significant better prognosis than the other two subgroups, and we found that NAL-H was related with significantly lower ITH and enriched in T helper cells. A recent paper reported that a high-ITH tumor had a strong immunosuppressive tumor microenvironment (Wolf et al., 2019). The relative expressions of the neoantigens in the high-ITH tumor were reduced, reducing the homing ability of TILs to their target cells and sufficient cytotoxic reactions. In our NAL-H subgroups, more neoantigens are exposed to the tumor microenvironment, which enhances the immune system’s antitumor ability. Meanwhile, with the higher T helper cells, it can further enhance the antitumor ability in NAL-H because CD4^+^ T cells can recognize MHC II antigen and work together with CD8^+^ T cells. These may be the reasons why NAL-H patients had better survival than did NAL-M patients.

We identified 30 SMGs among the identified subgroups, including 18 neoantigen gene sites of greater interest. The top five most common SMGs were TP53, PIK3CA, PTEN, ARID1A, and PIK3R1. Driver mutant genes (e.g., TP53, KRAS, and PIK3CA) may interfere with genomic stability and may affect the immune status by generating neoantigens (Markowitz and Bertagnolli, 2009). We also used 30 mutation signatures to better understand the complex mutational processes. Interestingly, we found that NAL-H was enriched in signature 6, which was more common in uterine tumors and related to DNA mismatch repair defects. Some studies have proven that mutant signature 6 may indicate that samples are sensitive to the immune inhibitors (Berger et al., 2018). NAL-H may be better responded to by ICI therapy.

To further explore the differences between NAL-H and NAL-M, we integrated gene expression and methylation data. Martens tretinoin response was up in NAL-M. Under physiological concentrations of tretinoin, PML-RARα has been reported to bind with RXR, which may be crucial to its carcinogenic potential (Martens et al., 2010). NAL-M may have more complex carcinogenic mechanisms. The ESR1 gene encodes an estrogen receptor, whose expression status distinguishes ER-positive from ER-negative tumors. A recent work showed differential methylation between these two distinct diseases (Dedeurwaerder et al., 2011). We found that a key CpG site, whose Illumina ID is cg17240454, was negatively related with the gene SPDEF. SPDEF is a member of the ETS family, whose high expression can promote the migration and invasion of various cells (Turner et al., 2007). The downregulation of SPDEF in NAL-M may stimulate the migration of cancer cells.

Our analyses were limited by the data form TCGA because pathologists excluded tumor samples with less than 60% tumor cell nucleus from the study and some important immune infiltrate samples might be lost (Berger et al., 2018). We also lacked experimental verification on the targeted classical cellular immunoassays for confirming cell phenotypic distribution. We hope that we could further understand the features of various NAL subgroups across Pan-Gyn cancers.

In summary, this comprehensive analysis of Pan-Gyn cancers revealed that 80% may be a good cutoff point for NAL across multiple tumor types. Among all samples, a higher NAL (highest 20% in each tumor) was associated with better overall survival. The obtained NAL subgroups were characterized by molecular features, immune signatures, and clinical outcome. As we increasingly recognize the impact of NAL on disease progression and treatment response, this biomarker may play an important role in predicting disease outcomes.

## Data Availability Statement

Publicly available datasets were analyzed in this study. This data can be found in The Cancer Genome Atlas Project (TGCA) pan-cancer analyses data portal (https://portal.gdc.cancer.gov/about-data/publications/pancanatlas).

## Author Contributions

FY, FW, and YZ contributed to the conception and design. YZ, XL, and XR developed the methodology. YZ and XL analyzed and interpreted the data. YZ and XR wrote, reviewed, and/or revised the manuscript. All authors contributed to the article and approved the submitted version.

## Conflict of Interest

The authors declare that the research was conducted in the absence of any commercial or financial relationships that could be construed as a potential conflict of interest.

## Footnotes

1 https://portal.gdc.cancer.gov/about-data/publications/pancanatlas

2 https://portal.gdc.cancer.gov/

3 http://www.firehose.org/

4 http://biopharm.zju.edu.cn/tsnadb/

5 http://hgdownload.cse.ucsc.edu/

## Supplementary Material

FIGURE S1 Overview of the MAF file.

FIGURE S2 Distribution of Pan-Gyn cancers and neoantigen load.

FIGURE S3 Response to chemotherapy among identified subgroups.

TABLE S1 Five statistically significantly dysregulate genes among identified subgroups.

TABLE S2 Gene Ontology annotation results of significantly dysregulate genes among identified subgroups.

TABLE S3 Thirty-seven genes highly related with AC092580.4.

TABLE S4 GSEA results for 37 genes highly related with AC092580.4.

TABLE S5 Thirty statistically significantly mutated genes among identified subgroups.

TABLE S6 GSEA results for differently expression genes between NAL-H and NAL-M.
