# Supplementary figures and images for: Characterization of Neoantigen Load Subgroups in Gynecologic and Breast Cancers
Source: Front Bioeng Biotechnol. 2020 Jul 13;8:702. doi: 10.3389/fbioe.2020.00702 (PMC7370692; doi:10.3389/fbioe.2020.00702)

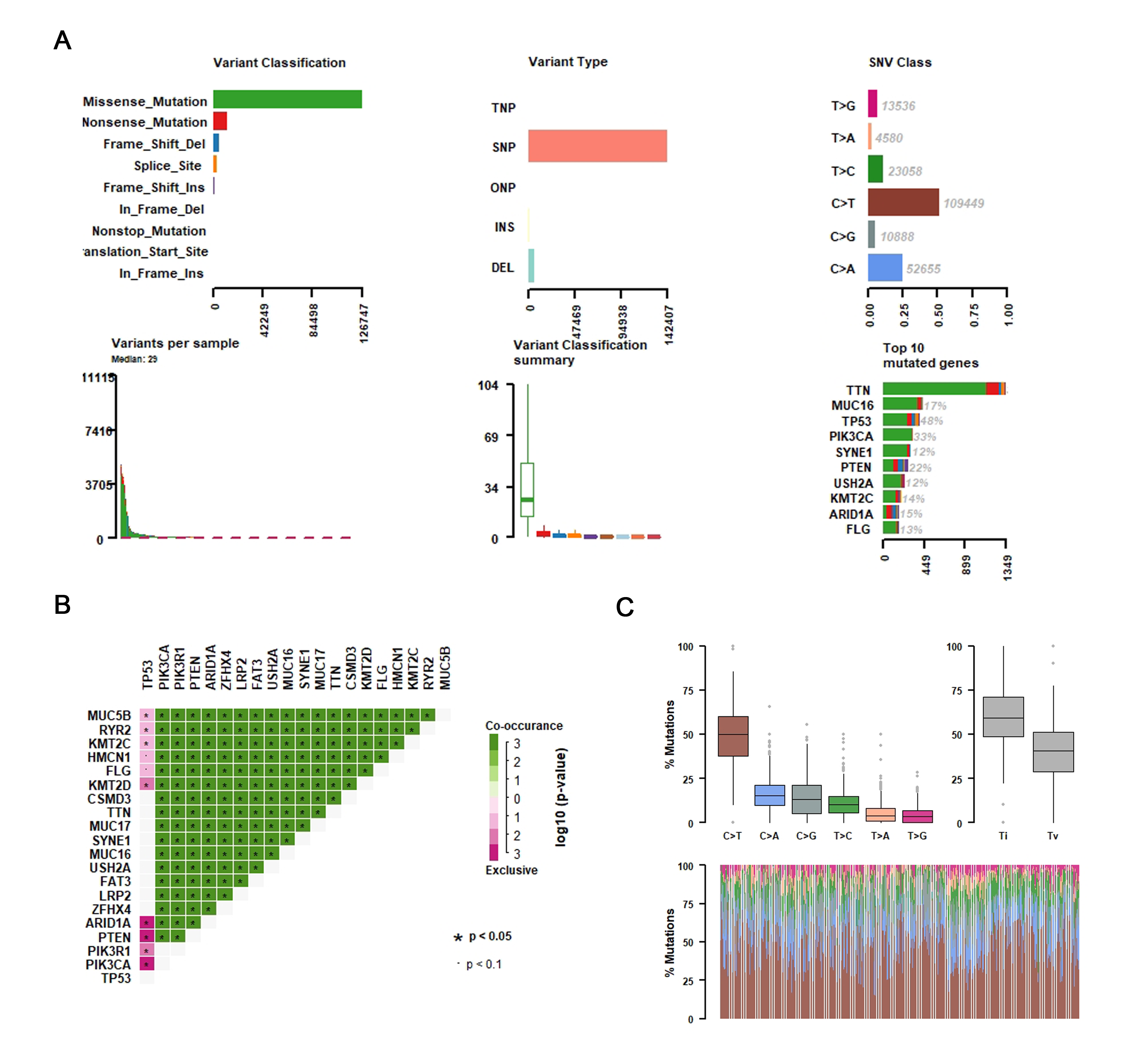

Supplement: FIGURE S1 — Overview of the MAF file. [file Image_1.TIF]

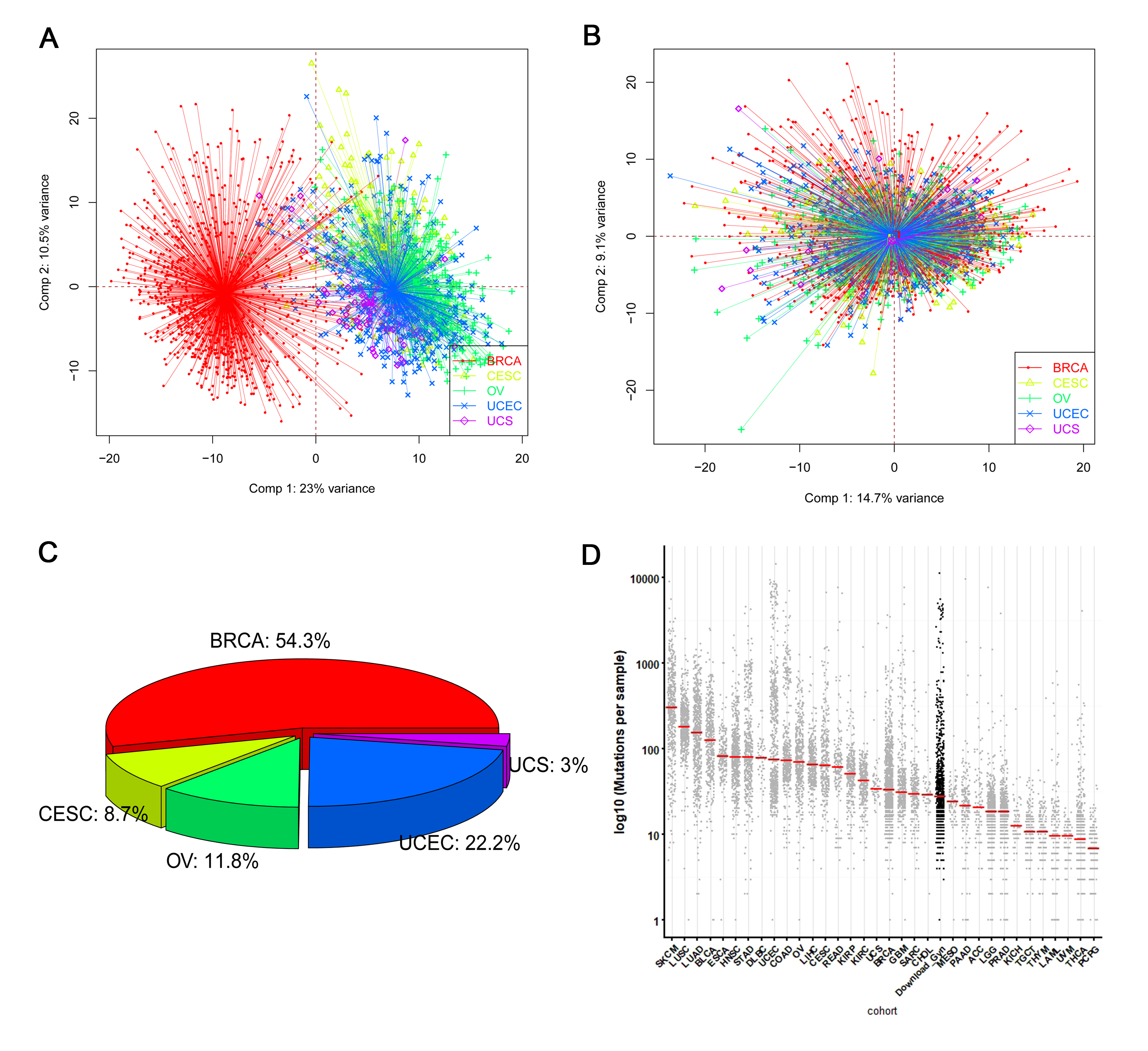

Supplement: FIGURE S2 — Distribution of Pan-Gyn cancers and neoantigen load. [file Image_2.TIF]

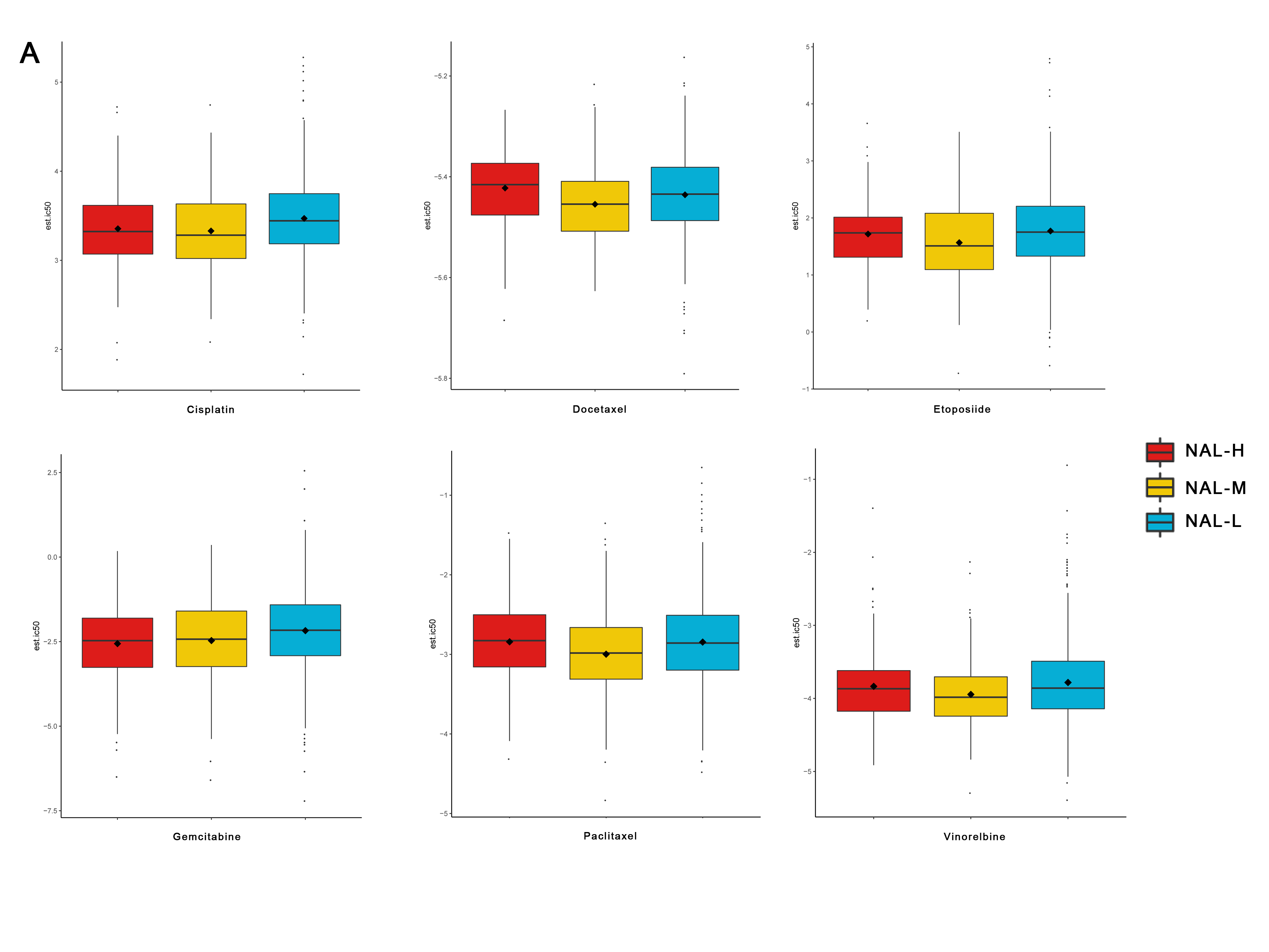

Supplement: FIGURE S3 — Response to chemotherapy among identified subgroups. [file Image_3.TIF]
